# Phytotoxic Action of Silver Nanoparticles on *Lemna minor*: Multi-Parameter Analysis of Different Physiological Processes

**DOI:** 10.3390/plants12020343

**Published:** 2023-01-11

**Authors:** Katarina Glavaš Ljubimir, Ana-Marija Domijan, Sandra Radić Brkanac

**Affiliations:** 1 Croatian Waters, Main Water Management Laboratory, 21 000 Split, Croatia; 2 Department of Pharmaceutical Botany, Faculty of Pharmacy and Biochemistry, University of Zagreb, 10 000 Zagreb, Croatia; 3 Department of Biology, Faculty of Science, University of Zagreb, 10 000 Zagreb, Croatia

**Keywords:** antioxidative enzyme, glutathione, malondialdehyde, nanosilver, photosystem II

## Abstract

Considering the widespread use of silver nanoparticles (AgNPs) and their consequent build-up in waterways, there is a concern about the hazardous effect of AgNPs for aquatic ecosystems. The aim of this study was to clarify the mechanism of the action of AgNPs on duckweed (*Lemna minor* L.) by evaluating multiple parameters in different physiological processes. Duckweed was treated with AgNPs in a concentration range of 0.5 to 5 mg/L over a 7-day period. The analysis revealed that the AgNP-treated duckweed accumulated Ag in accordance with increasing AgNP concentrations. Furthermore, higher concentrations (2 and 5 mg/L) of AgNPs negatively affected N, P and especially K and Mg levels in the plant tissue. Accordingly, the plant growth and photosynthetic parameters were more inhibited in response to higher concentrations of AgNPs. Nanosilver significantly increased the generation of ROS at higher concentrations, although lipid peroxidation was significant even at the lowest concentration of AgNPs. However, defense mechanisms were able to counteract AgNP-induced oxidative stress and balance the intracellular redox status, as evidenced by increased activities of the main detoxification enzymes. With this experimental setting, AgNPs exhibited a relatively weak phytotoxicity at 0.5 and 1 mg/L; nevertheless, silver in a nano form poses a hazard for plants, considering its continuous release into aquatic environments.

## 1. Introduction

Nanoparticles (NPs) have applications in various fields of science and technology as well as in many consumer products, including electronic components, cosmetics, food products, cleaning products, antimicrobial fibers and sprays. Silver nanoparticles (AgNPs) are the most commonly used nanomaterial in various fields, especially in agriculture [1].

Due to their extensive use, an increasing amount of AgNPs ends up in surface waters, with the potential to have adverse effects on aquatic ecosystems [2,3]. Once in the environment, AgNPs undergo a loss and change of the surface coating, aggregation and agglomeration as well as surface oxidation and the release of silver ions (Ag^+^) [4,5]. Free Ag^+^, which may be released from AgNPs, has generally been recognized as one of the most toxic forms among the heavy metals in aqueous environments [6]. However, the phytotoxicity cannot be explained only by the released Ag^+^ because several studies have found that AgNPs are more toxic than Ag+ at the same concentration [7]. A number of studies have revealed that it is difficult to determine which part of the toxicity can be attributed to the nano form and which to the ionic form of Ag [2,8,9].

The increased use of AgNPs in consumer products, the likelihood of their release into the environment [10] and the demonstrated adverse effect on aquatic organisms justifies research into the phytotoxicity of AgNPs [3]. Here, a model plant species, *Lemna minor* L., was selected. Duckweed (*L. minor*) is a freshwater macrophyte known for its large reproductive capacity, fast growth, small size and relatively simple structure as well as easy cultivation. Due to these desirable traits, duckweed is often used in studies on the impact of heavy metals in aquatic environments [11]. In this research, the toxicity of test substances to duckweed was assessed according to the OECD Guidelines, Test No. 221 [12].

AgNPs have been evidenced to exert a plethora of negative effects on the physiological level of the nutrient status as well as growth and photosynthesis [4,13,14]. The performance of the latter process in plants exposed to nanosilver was reported to result in modifications to the chloroplast structure [15], in a reduction in chlorophylls and carotenoids [16,17] and in a decrease in chlorophyll *a* fluorescence parameters such as the maximum (Fv/Fm) and effective (*Φ*PSII) quantum yield of photosystem II (PSII) [9,18]. Moreover, it seems that AgNP-driven phytotoxicity is tightly connected to the disruption of the cellular redox homeostasis, which leads to an increased production of reactive oxygen species (ROS) and consequent oxidative damage to cellular components, including cell membranes and DNA as well as the activation of antioxidant enzymes, the exhaustion of antioxidant molecules, protein oxidative modifications and their inactivation [7,19].

However, existing studies have often focused on one aspect of nanosilver-induced phytotoxicity, giving fragmentary data about the mechanism of the toxic action of AgNPs in aquatic plants. In the present study, we aimed to discern the mode of phytotoxic action of nanosilver, integrating data from multiple parameters to evaluate the growth, nutrient status, photosynthetic performance, oxidative stress and plant defense mechanisms in one experimental setting. We used AgNPs coated with polyvinylpyrrolidone (PVP) because PVP, along with citrate, is a dominant capping agent in commercial products; more importantly, in freshwater and seawater, PVP-coated AgNPs have been shown to be more stable than AgNPs coated with other shields [3]. In this experimental setting, duckweed was treated with AgNPs in concentrations of 0.5, 1, 2 and 5 mg/L for a 7-day period. Those concentrations were selected based on our preliminary results and on previous studies [20].

## 2. Results and Discussion

### 2.1. Impact of AgNPs and AgNO_3_ on Ag Accumulation

In comparison with existing studies, we used an integrative approach to evaluate and compare the impact of nanosilver on multiple parameters in different physiological processes (such as the growth, nutrient status, photosynthesis and oxidative stress) in duckweed.

**Figure 1:**
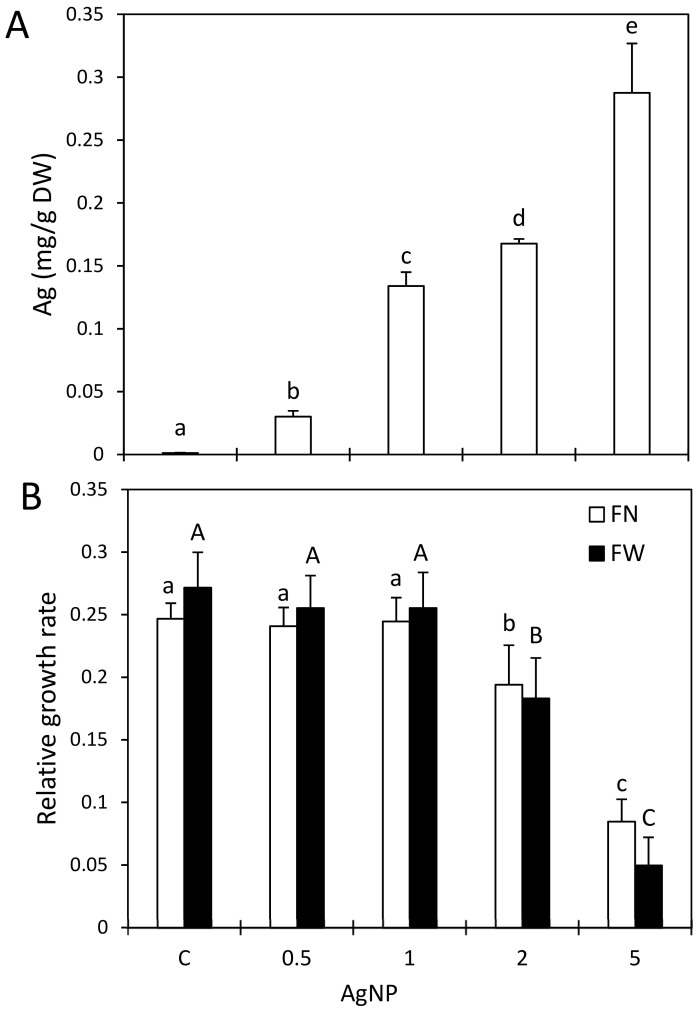
Ag content (**A**) and FN- or FW-based relative growth rate (RGR) (**B**) in duckweed after a 7-day treatment with silver nanoparticles (AgNPs) in a concentration range of 0.5–5 mg/L or in control plants (C). Standard deviations are presented by error bars. Different letters indicate significantly different values at *p* < 0.05 according to an ANOVA.

The accumulation rate of Ag in the AgNP-treated duckweed is presented in Figure 1A. With the increase in the concentrations of AgNPs in the nutrient medium, a linear increase in the Ag accumulation rate in the plant tissue was observed. As it is known that nanosilver toxicity is partially driven by Ag^+^, Ag accumulation was also evaluated in AgNO_3_-treated duckweed under the same experimental conditions for comparison. In the plants treated with AgNO_3_, a significantly higher level of Ag was observed in comparison with the treatment with AgNPs (Figure S1). Studies so far have demonstrated that plants accumulate Ag more readily when exposed to its ionic form [5,9,19]. A lower accumulation of Ag in the plant tissue after a treatment with AgNPs compared with one with AgNO_3_ might be due to a weaker dissolution of AgNPs and/or the formation of agglomerates, leading to a lower concentration of released Ag^+^ and a slower uptake of AgNPs/Ag^+^ by the plant cells [15].

### 2.2. Impact of AgNPs on Growth and Selected Nutrients

The 7-day exposure of duckweed to higher concentrations of AgNPs caused morphological changes (separated colonies, damaged roots and leaf necrosis), indicating a negative effect of Ag on duckweed growth. Lower concentrations of AgNPs did not affect the growth whilst higher concentrations (2 and 5 mg/L) caused a significant reduction in the duckweed growth rate (Figure 1B). A reduction in growth due to AgNPs of different sizes and concentrations has been reported in aquatic and terrestrial plants [3,8,9,15,21,22,23,24]. The decreased growth rate of duckweed after the AgNP treatment may have been connected to the accumulation of Ag in the plant cells and consequent changes in the nutrient contents (Table 1). The Ag content showed a very strong negative correlation with K and Mg as well as a strong negative correlation with N and the growth parameters (Table S1).

Nanosilver showed the strongest effect on K and Mg contents because a significant decrease in those nutrients in the AgNP-treated duckweed compared with the control had already been recorded from 1 mg/L (Table 1). The content of N in the plants significantly decreased in response to higher AgNP concentrations whereas that of P was negatively affected only in response to 5 mg/L. It is interesting that the P and N contents showed a strong negative correlation with the ROS, suggesting that the modulation of those nutrients was linked to a redox imbalance (Table S1).

A negative influence of AgNPs on Ca, Mg, P, Cu, Mn and Zn was previously noted in radish (*Raphanus sativus*) seedlings [25]. In that study, it was assumed that nanosilver might have affected the expression of the metal transporters or potentially physically blocked the channels, thus reducing the absorption of Ca and Mg and other nutrients.

However, in our study, the Ca content increased in the plants treated with higher concentrations of AgNPs (the increase was significant at 5 mg/L compared with the control). Such results might be explained by the action of Ag^+^ on calcium channels, leading to a transient increase in cytosolic Ca [26]. Those authors inferred that Ag^+^ via a modification of the Ca levels disturbed the membrane integrity in tobacco BY-2 cells, influencing the level of ions and phytohormones, which could, in turn, affect the plant growth.

### 2.3. Impact of AgNPs on Photosynthetic Pigments and Photosynthesis

Similar to the growth rate, only the higher concentrations of AgNPs significantly reduced the level of chlorophylls and carotenoids compared with the control (Table 2).

A decrease in the level of photosynthetic pigments after exposure to AgNPs has been recorded in mustard seedlings [9], duckweed species [17,19,21] and in tobacco [27]. Evidence shows that heavy metals, in general, negatively affect chlorophylls synthesis by a substitution of Mg^2+^ with heavy metal ions in the chlorophyll molecule, by chloroplast membrane degradation due to lipid peroxidation or by a reduced antioxidant concentration [28].

Regarding the chlorophyll *a* fluorescence parameters, only higher concentrations of AgNPs significantly reduced Fv/Fm and *Φ*PSII in the duckweed fronds whereas the lower concentrations did not change those parameters (Table 2). At the same time, higher concentrations of AgNPs increased NPQ, although the increase was significant only in response to the highest AgNP treatment (Table 2). An increase in NPQ and an inhibition of PSII photochemistry in response to an AgNP treatment have also been noted in the aquatic macrophyte *Spirodela polyrhiza* [29]. In that study, the negative impact of Ag on the photosynthetic processes correlated with an increase in its concentration. Such results were similar to ours, as a strong negative correlation of the level of Ag in the plant tissue and the PSII parameters was observed (Table S1). A reduction in *Φ*PSII and an electron transfer from plastocyanin to PSI may occur due to the competitive substitution of Cu in plastocyanin with Ag [30]. On the other hand, increased heat dissipation could have a protective role against an over-reduction of the electron transport chain in the conditions of reduced CO_2_ assimilation and thus a reduced requirement for NADPH and ATP [31].

In the present study, the PSII parameters showed a strong negative correlation with Ag and the parameters of oxidative damage (Table S1). Such results suggested that a decrease in the PSII photochemical reactions might have arisen by the Ag-induced formation of lipid peroxides in the thylakoid membranes of the chloroplasts. In addition, the PSII parameters positively correlated with the GSH, indicating an important role of that antioxidant on the stability of PSII (Table S1).

### 2.4. Impact of AgNPs on Cellular Redox Balance

**Figure 2:**
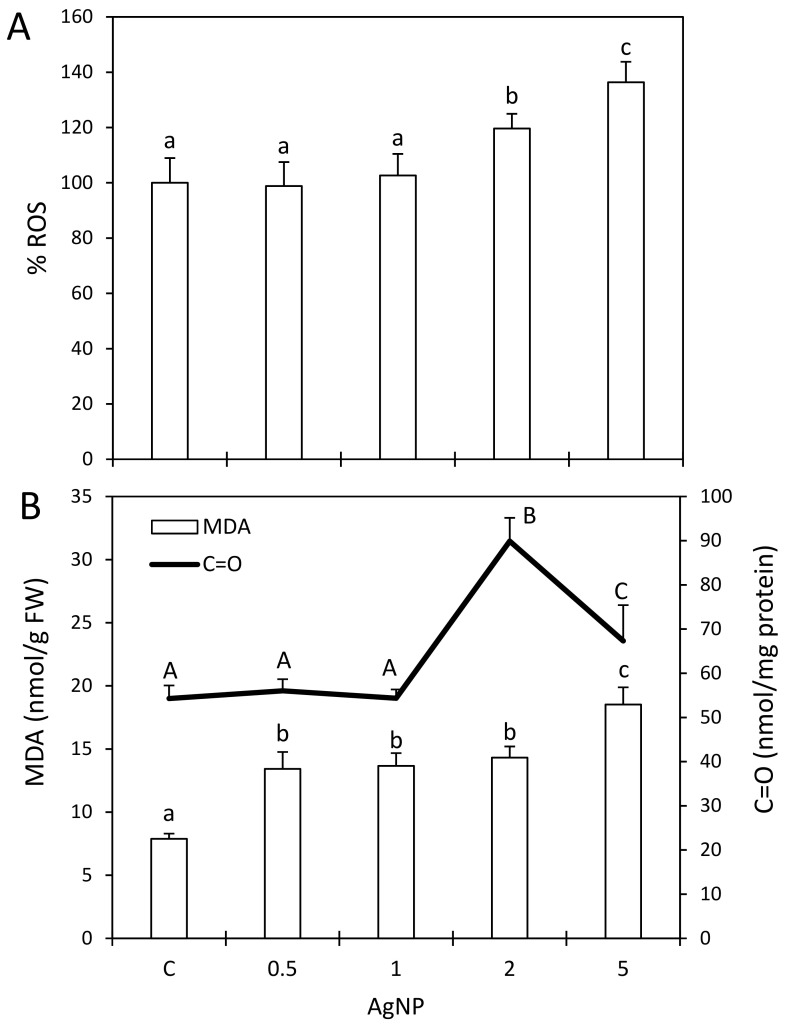
Reactive oxygen species (ROS) (**A**), malondialdehyde (MDA) and carbonyls (C=O) (**B**) in duckweed after a 7-day treatment with silver nanoparticles (AgNPs) in a concentration range of 0.5–5 mg/L or in control plants (C). Standard deviations are presented by error bars. Different letters indicate significantly different values at *p* < 0.05 according to an ANOVA.

Higher concentrations of AgNPs caused a significant increase in the ROS (10–36% compared with the control) in the plant cells (Figure 2A). However, the MDA level (biomarker of lipid peroxidation) was significantly elevated regardless of the concentration of AgNPs (Figure 2B). An observed discrepancy between the ROS and MDA levels might be explained by the fact that the fluorescent probe (DHE) used in this study for the detection of ROS mostly detects superoxide radicals. Thus, a greater extent of lipid peroxidation in response to AgNPs might have been due to the involvement of other ROS such as hydroxyl radicals.

The biomarker of oxidative damage to proteins, expressed as protein carbonyls (C=O), only increased in the duckweed treated with higher concentrations (2 and 5 mg/L) of AgNPs whereas lower concentrations of nanoparticles had no effect on that parameter (Figure 2B). Similar to this study, several other reports have confirmed a significant increase in ROS, MDA and protein carbonylation in different plant species following treatments with AgNPs [4,19,21].

To counteract the detrimental action of excess ROS on various cell structures and biomolecules, plant cells activate different sets of antioxidative defenses, which comprise enzymatic and non-enzymatic molecules [7]. Here, the activities of the main antioxidant enzymes—SOD, CAT and APX—were induced by AgNPs (Table 3).

The latter increased the SOD activity in the duckweed at all applied concentrations and the highest increase in SOD activity (38% compared with the control) was recorded in the plants treated with 2 mg/L of AgNPs (Table 3). Hydrogen peroxide generated by SOD can be efficiently detoxified by the action of CAT and APX. Both enzymes increased after the AgNP treatments (except under the lowest concentration) compared with the control. The enzymes SOD and CAT showed a strong mutual correlation (Table S1), but a strong correlation between SOD and CAT and carbonyls (Table S1) was also established, indicating the activation of those enzymes under severe oxidative damage to the proteins.

Data show that AgNPs of different coatings and size mostly stimulate SOD and H_2_O_2_-detoxifying enzyme activities in both aquatic [20] and terrestrial [18] plant species, corroborating the results of our study.

In addition to APX, we also evaluated the activity of non-specific peroxidase (GPX) that is not present in the chloroplasts, mitochondria and peroxisomes; it is involved not only in the breakdown of H_2_O_2_, but also in cell wall cross-linking, suberin and lignin formation, senescence, the metabolism of auxin (IAA) and the defense against abiotic and biotic stress conditions [32]. In our study, the activity of GPX in plants treated with almost all applied concentrations of AgNPs significantly increased compared with the control (Table 3). The induction of GPX was consistent with the accumulation of Ag in the duckweed, as indicated by a very strong correlation between the GPX and Ag levels (Table S1). In addition, GPX showed an inverse correlation with the growth and photosynthetic parameters (Table S1). A number of studies have reported that an increase in non-specific peroxidase in response to heavy metals is associated with enhanced ROS and lignin formation and a consequent growth reduction [33,34]. An observed stiffening of the cell wall is regarded as a defense reaction that limits the uncontrolled entry of toxic metals in plant cells [33,34]. Increased GPX activity and simultaneous growth inhibition have been recorded in AgNP-treated duckweed [8].

Studies have demonstrated that the GST activity usually increases in plants after heavy metal exposure, although the enzyme has a vital role in the flavonoid metabolism, growth and detoxification of xenobiotics and toxic lipid peroxides formed during oxidative stress [35]. Here, the GST activity significantly increased in the plants treated with AgNPs (at concentrations higher than 0.5 mg/L) (Table 3). An inverse correlation between GST and GSH (Table S1) implicated the use of GSH as a substrate in a GST-mediated reaction. According to the correlation between GPX and GSH (Table S1), it seemed that GSH was consumed as a substrate by GPX as well [8]. The level of that antioxidant in the duckweed treated with AgNPs at concentrations higher than 0.5 mg/L significantly decreased compared with the control (Table 3). The observed negative correlations of GSH with Ag and positive correlations of GSH with the photosynthetic parameters (Table S1) indicated the depletion of the GSH pool in the detoxification of the ROS, but also indicated the vital role of that antioxidant in maintaining the redox homeostasis [35].

## 3. Materials and Methods

### 3.1. Chemicals and Characterization of AgNPs in a Nutrient Medium

Commercially available PVP-coated AgNPs were obtained from Sigma-Aldrich (St. Louis, MO, USA). Detailed information on the characteristics of AgNPs is provided in our previous study [20]. The same batch of AgNPs was used in this study. In short, the AgNPs were spherical and formed small agglomerates in a Steinberg medium. According to TEM, the particle size was 45.78 ± 7.46 nm and the diameter of the particle size distribution was 162.7 ± 12.6 nm (according to dynamic light scattering). AgNO_3_ was procured from Sigma-Aldrich (St. Louis, MO, USA). All other chemicals were obtained from Sigma-Aldrich as well, unless stated otherwise.

### 3.2. Plant Material and Experimental Design

Duckweed (*Lemna minor* L.) plants were maintained as stock cultures on a Pirson–Seidel nutrient medium with a pH of 4.55 [36]. Prior to the experiments, the plants were adapted to the Steinberg nutrient medium with a pH of 5.5 [12] for a 7-day period. Following the 7-day adaptation period, several healthy colonies from the stock cultures were transferred to Steinberg nutrient media, to which AgNPs or AgNO_3_ were added to final concentrations of 0.5 mg/L, 1 mg/L, 2 mg/L and 5 mg/L. Duckweed grown on the Steinberg medium served as a control (C; control media). The exposure period of the AgNP-treated, AgNO_3_-treated or controls was 7 days. The plants were grown under a 16 h photoperiod of fluorescent light (90 mEm^−2^ s^−1^) at 24 ± 1 °C. Prior to use, both nutrient media (Pirson–Seidel or Steinberg) were autoclaved at 0.15 MPa at 120 °C for 20 min.

### 3.3. Level of Ag and Selected Nutrients in Plant Material

To determine the concentration of Ag, K, Ca, Mg, N and P, the plant material was dried until a constant weight was obtained (usually 24 h at 80 °C). For the determination of Ag, K, Ca and Mg, approximately 0.5 g of dried plant material in Teflon cuvettes was digested by adding 6 mL of Suprapur HNO_3_; afterwards, the samples were heated for half an hour at 1000 W (Anton PaarMultiwave 3000 Oven, Anton Paar GmbH, Graz, Austria). The dissolved samples were quantitatively transferred to volumetric flasks and diluted to 50 mL with deionized water. The concentration of Ag, K, Ca and Mg was determined by inductively coupled plasma mass spectrometry (ICP-MS, Elan 9000, PerkinElmer, Inc., Waltham, MA, USA) using 20 mg/L Ge, Rh, In and Re as internal standards, according to HRN EN ISO 17294e1 and HRN EN ISO 17294e2 norms. Calibration curves of Ag and the internal standards were obtained by using a PerkinElmer multi-element calibration standard solution (PerkinElmer, USA). The measurements were performed in triplicate and the relative standard deviation (RSD) was better than 15%. The quality control of the ICP-MS method was performed by the analysis of Ag in standard reference material from Council Canada (TM-RAIN04 and BURTAP-05).

The contents of total N and P in the plant material were determined spectrophotometrically according to ISO/TR 11,905 [37] and ISO 6878 [38].

### 3.4. Toxicity Parameters: Growth Rate

The growth of the duckweed was estimated by assessing two parameters (x): the frond number (FN) and the fresh weight (FW, biomass). Those parameters were scored at the beginning of the experiments (t0) and after a 7-day exposure (t1). The FW of the plants was determined after the surface-drying of the plants between layers of paper towels. From the measured parameters, the relative growth rate (RGR) was calculated separately according to the following equation for each replicate:

$$\mathrm{RGR}=\frac{\ln x\;t1-\ln x\;t0}{t1-\;t0}$$

### 3.5. Measurement of Photosynthetic Pigments and Chlorophyll a Fluorescence

Chlorophyll *a* fluorescence in situ was measured by the saturation pulse method using a “Qubit” fluorescence measurement system; all parameters were calculated according to Maxwell and Johnson [39]. Prior to the measurement, the plants (five fronds per each replicate) were kept in darkness for 30 min and the minimum fluorescence yield (F0) was measured. The maximum fluorescence yield of the dark-adapted fronds (Fm) was then measured after a short pulse of saturating light. From these data, the maximum quantum yield of photosystem II, Fv/Fm, was calculated. The maximum fluorescence yield of the light-adapted fronds (F’m) and the steady-state fluorescence (F) were measured after the application of light of 100 µmol/m^2^/s for 15 min. Those values were used to calculate the effective quantum yield of PSII (*Φ*PSII) and non-photochemical quenching (NPQ).

Chlorophylls and carotenoids were measured and their concentrations calculated according to Lichtenthaler [40]. Briefly, 30 mg of fresh tissue samples was homogenized with 80% (*w*/*v*) cold acetone, centrifuged at 5000× *g* for 10 min and the absorbances of the supernatant were read at 663, 646 and 470 nm.

### 3.6. Oxidative Stress Parameters

The levels of lipid peroxidation and protein oxidation were assessed via malondialdehyde (MDA) and protein carbonyl contents, respectively [20]. In short, for the lipid peroxidation, the plant tissue was homogenized with 0.25% (*w*/*v*) of a thiobarbituric (TBA) acid solution containing 10% (*w*/*v*) trichloroacetic acid and then heated at 95 °C for 30 min. Afterwards, the reaction mixture was cooled and centrifuged. The MDA-TBA content (nmol/g FW) was measured at 532 nm and calculated by using the extinction coefficient of 0.155 L/mol/cm. The amount of protein carbonyls (C=O) was estimated by the reaction of the carbonyl groups with 2,4-dinitrophenylhydrazine (DNPH). After the DNPH reaction, the carbonyl content was calculated by absorbance at 370 nm using an extinction coefficient for aliphatic hydrazones of 0.022 L/mol/cmand this was expressed as nmol carbonyl/mg protein.

For the assessment of the antioxidant enzyme activities, the plant tissue was homogenized in 50 mmol/L of a K_2_HPO^4^/KH_2_PO_4_ buffer (pH 7.0) containing 0.1 mmol/L ethylene diamine tetraacetic acid (EDTA). The homogenates were centrifuged at 25,000 × *g* for 30 min at 4 °C and the supernatants were used for the determination of the protein content and enzyme activities. The total soluble protein concentration of the plant extracts was estimated using bovine albumin serum as a standard at 595 nm. The superoxide dismutase (SOD) activity was determined according to Giannopolitis and Ries [41]. The reaction solution contained 50 mmol/L of a potassium phosphate buffer (pH 7.8), 13 mmol/L methionine, 75 µmol/L nitrotetrazolium blue chloride salt (NBT), 0.1 mmol/L EDTA, 2 µmol/L riboflavin and a sample supernatant. Riboflavin (10 µL) was added to the reaction mixture immediately prior to the measurement. The sample was mixed and placed under a light source (15 W) in a darkened room. The reaction was initiated by the inclusion of light (the photoreactive riboflavin initiated the formation of superoxide radicals) and after 10 min of measurement the light was turned off. NBT is reduced in the presence of superoxide radicals in an insoluble blue-colored formazan, showing an absorption maximum at a wavelength of 560 nm. One unit of SOD was taken as the volume of the enzyme extract causing a 50% inhibition of the NBT reduction.

The catalase (CAT) activity was determined by a decrease in the H_2_O_2_ concentration and was measured by following the decrease in absorbance at 240 nm [42]. The activity was calculated using the molar extinction coefficient of 0.040 L/mol/cm and µmol H_2_O_2_ decomposed/g FW/min was defined as the unit of CAT. The ascorbate peroxidase (APX) activity was determined according to Nakano and Asada [43]. The ascorbate oxidation was followed at 290 nm and its concentration was calculated using the molar extinction coefficient of 2.8 L/mmol/cm. One enzyme unit was defined as µmol oxidized ascorbate/g FW/min. The reaction solution for the determination of the guaiacol peroxidase (GPX) activity contained 50 mmol/L of a potassium phosphate buffer (pH 7.0), 18 mmol/L guaiacol and 5 mmol/L H_2_O_2_. The increase in the absorbance due to tetraguaiacol formation was measured at 470 nm [44] and GPX was calculated using the extinction coefficient of 26.6 L/mmol/cm.

The reaction solution for determining the glutathione-S-transferase (GST) activity contained 100 mmol/L of a potassium phosphate buffer (pH 6.5), 10 mmol/L of reduced glutathione, 1 mmol/L EDTA, 100 mmol/L 1-chloro-2,4-dintrobenzene (CDNB) and a sample supernatant [45]. The increase in absorbance due to the reduction of the CDNB substrate by the SH group of glutathione was monitored at a wavelength of 340 nm. The GST activity was calculated using the appropriate molar extinction coefficient (ε340 = 9.6 L/mmol/cm).

The specific enzyme activity for all enzymes was expressed as units/mg protein.

The level of reactive oxygen species (ROS) and glutathione (GSH) in duckweed was determined using the fluorescent probes dihydroethidium (DHE) and monochlorobimane (MCB), respectively [20]. After the treatment, to assess the ROS level, the plant (duckweed) was incubated with 10 µL of 10 µmol/L DHE for 30 min at room temperature and protected from light. DHE is a membrane-permeable compound that is oxidized to a red fluorescent ethidium mostly by the action of superoxide radicals. To assess GSH, after the treatment, the duckweed was incubated with 10 µL of 10 µmol/L MCB at room temperature and protected from light. MCB forms a fluorescent adduct with GSH; therefore, an increase in fluorescence is suggestive of an increase in the GSH level. The slides were examined under an Olympus BX-51 epifluorescent microscope connected to a camera (Olympus DP70, Tokyo, Japan) at an excitation of 450–490 nm and an emission of 520 nm or greater and an excitation of 380 nm and an emission of >400 nm for the ROS and GSH assays, respectively. Images of the fluorescence were analyzed using software (Lucida 6.0, Wirral, UK). A total number of at least 100 cells was counted in 10 fields of each slide. Identical conditions were maintained in all the experimental groups.

### 3.7. Statistical Analysis

Data were presented as the mean ± standard deviation. Each data point was the average of six replicates (*n* = 6), with the exception of the ICP-MS analysis and the chlorophyll a fluorescence, which were performed with three replicates (*n* = 3). The statistical analysis was performed by STATISTICA 12.0 (StatSoft, Inc., Tulsa, OK, USA). The normality of the data was tested by Shapiro–Wilk’s W-test. The homogeneity of variance for each dependent variable was tested by Levene’s test. The possible difference among the samples was assessed by a one-way ANOVA followed by a Duncan post hoc comparison test. In all the statistical tests, the significance level was set to *p* < 0.05. Different letters indicated significant differences between the treatments for each variable. Pearson’s correlation (r) coefficients between the different parameters were calculated.

## 4. Conclusions

**Figure 3:**
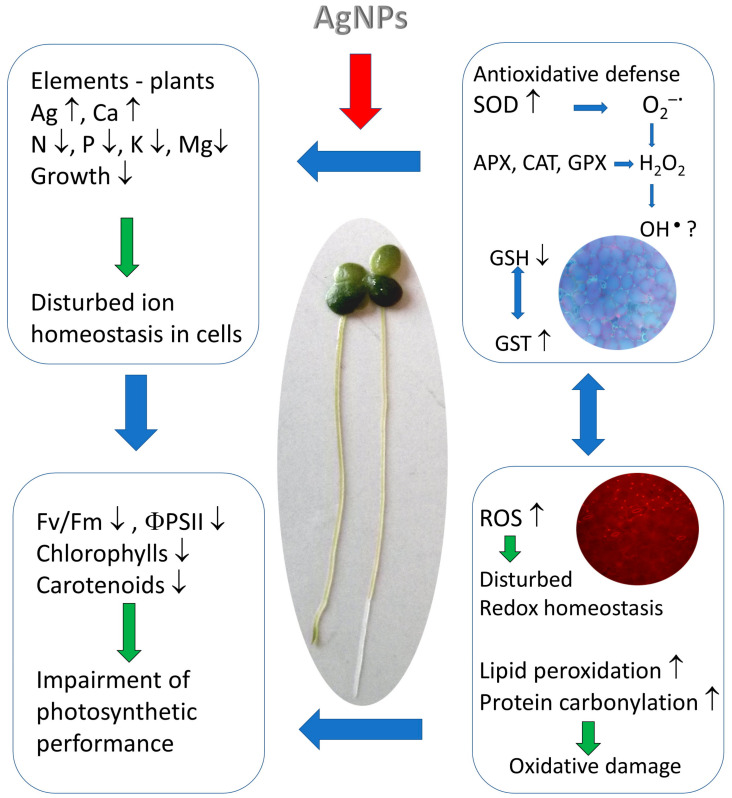
Schematic depiction of putative action of AgNPs in plant cells.

In comparison with existing studies, an integrative approach was used to evaluate and compare the impact of nanosilver on multiple parameters in different physiological processes (such as the growth, nutrient status, photosynthesis and oxidative stress) in duckweed. Overall, it was concluded that the mechanism of phytotoxicity of AgNPs involved: (1) an increased generation of ROS, leading to impaired cellular redox homeostasis and oxidative damage; and (2) the modification of nutrient levels, probably due to the effect of Ag on the ion channels, which, in turn, negatively affected photosynthesis and suppressed the duckweed growth (Figure 3).

Although less toxic at lower applied concentrations, it is evident that nanosilver is harmful to aquatic plants even with the relatively short exposure specified by the OECD test guidelines. Future studies on AgNP toxicity should, therefore, consider prolonged exposure periods of duckweed in the recommended media, but also in aqueous environmental matrices—e.g., surface waters under the same experimental setting—thus providing an adequate comparison and a realistic assessment of the environmental impact of AgNPs.

## Figures and Tables

**Table 1:** Macronutrient levels (mg/g DW) in duckweed after a 7-day treatment with different concentrations of silver nanoparticles (AgNPs) or in control plants (C).

mg/L	N	P	K	Mg	Ca
C	41.68 (1.10) ^a^	22.80 (1.25) ^a^	54.10 (0.94) ^a^	2.58 (0.058) ^a^	13.94 (0.54) ^b^
AgNP					
0.5	40.11 (0.82) ^ab^	20.72 (1.57) ^a^	54.01 (4.86) ^a^	2.60 (0.042) ^a^	14.82 (0.69) ^b^
1	40.26 (0.55) ^ab^	23.20 (1.72) ^ab^	46.67 (3.16) ^b^	2.31 (0.043) ^b^	14.77 (0.23) ^b^
2	38.65 (1.45) ^b^	21.38 (1.77) ^ab^	46.55 (3.26) ^b^	2.33 (0.154) ^b^	15.11 (0.85) ^ab^
5	39.48 (0.44) ^b^	18.87 (2.96) ^b^	41.18 (2.12) ^b^	2.25 (0.079) ^b^	16.21 (0.92) ^a^

Values represent the mean of three replicates (SD in parenthesis). Different letters within each column indicate a significant difference at *p* < 0.05 according to an ANOVA.

**Table 2:** Chlorophyll *a* fluorescence parameters and photosynthetic pigments (mg/g FW) in duckweed after a 7-day treatment with different concentrations of silver nanoparticles (AgNPs) or in control plants (C).

mg/L	Fv/Fm	*Φ*_PSII_	NPQ	Chl *a*	Chl *b*	Car
C	0.67 (0.009) ^a^	0.58 (0.017) ^a^	0.061 (0.011) ^b^	0.71 (0.022) ^a^	0.27 (0.014) ^a^	0.29 (0.008) ^a^
AgNP						
0.5	0.66 (0.011) ^a^	0.56 (0.015) ^a^	0.053 (0.007) ^b^	0.70 (0.019) ^a^	0.28 (0.008) ^a^	0.29 (0.008) ^a^
1	0.66 (0.009) ^a^	0.56 (0.017) ^ab^	0.052 (0.017) ^b^	0.69 (0.006) ^a^	0.26 (0.005) ^a^	0.28 (0.010) ^a^
2	0.64 (0.009) ^b^	0.55 (0.016) ^b^	0.073 (0.008) ^ab^	0.63 (0.020) ^b^	0.24 (0.014) ^b^	0.26 (0.017) ^b^
5	0.63 (0.009) ^b^	0.54 (0.017) ^b^	0.088 (0.011) ^a^	0.62 (0.014) ^b^	0.22 (0.024) ^c^	0.24 (0.023) ^c^

Values represent the mean of three (chlorophyll *a* fluorescence) or six (chlorophyll and carotenoid) replicates (SD in parenthesis). Different letters within each column indicate a significant difference at *p* < 0.05 according to an ANOVA. Fv/Fm: maximum quantum yield of photosystem II; *Φ*PSII: effective quantum yield of PSII; NPQ: non-photochemical quenching; Chl *a*: chlorophyll *a*; Chl *b*: chlorophyll *b*; Car: carotenoids.

**Table 3:** Antioxidative enzyme activity (U/mg protein) and glutathione level (GSH, %) in duckweed after a 7-day treatment with different concentrations of silver nanoparticles (AgNPs) or in control plants (C).

mg/L	SOD	APX	CAT	GPX	GST	GSH
C	15.1 (0.77) ^c^	0.97 (0.064) ^b^	0.15 (0.008) ^c^	0.40 (0.023) ^c^	0.27 (0.013) ^c^	100.0 (5.31) ^a^
AgNP						
0.5	17.4 (0.51) ^b^	0.96 (0.072) ^b^	0.17 (0.009) ^bc^	0.46 (0.041) ^c^	0.29 (0.021) ^c^	94.0 (3.84) ^a^
1	15.9 (0.72) ^c^	1.14 (0.037) ^a^	0.17 (0.008) ^b^	0.60 (0.028) ^b^	0.34 (0.006) ^b^	86.4 (4.22) ^b^
2	20.8 (1.62) ^a^	1.12 (0.096) ^a^	0.24 (0.008) ^a^	0.57 (0.033) ^b^	0.38 (0.051) ^b^	84.0 (2.71) ^b^
5	19.5 (1.30) ^a^	1.13 (0.052) ^a^	0.25 (0.010) ^a^	0.72 (0.082) ^a^	0.45 (0.035) ^a^	82.2 (2.03) ^b^

Values represent the mean of six replicates (SD in parenthesis). Different letters within each column indicate a significant difference at *p* < 0.05 according to an ANOVA. SOD: superoxide dismutase; APX: ascorbate peroxidase; CAT: catalase; GPX: guaiacol peroxidase; GST: glutathione-S-transferase; GSH: glutathione.

## Data Availability

The data presented in this study are available on request from the corresponding author (S.R.B).

## Supplementary Materials

The following supporting information can be downloaded at: https://www.mdpi.com/article/10.3390/plants12020343/s1, Table S1: Pearson’s coefficient of correlation between parameters; Figure S1: Ag content in duckweed after a 7-day treatment with silver nanoparticles and ionic silver.
