# Supplementary material for: Phytotoxic Action of Silver Nanoparticles on Lemna minor: Multi-Parameter Analysis of Different Physiological Processes
Source: Plants (Basel). 2023 Jan 11;12(2):343. doi: 10.3390/plants12020343 (PMC9861787; doi:10.3390/plants12020343)
Supplement: Supplementary file 1 [file plants-12-00343-s001.zip › plants-1962785-supplementary.pdf]

Table S1. Pearson's coefficient of correlation between parameters (r values).

	Ag	K	Mg	P	N	Ca	RGR fr	RGR FW	Fv/Fm	Φ_{PSII}	NPQ	Chl a	Chl b	Car	MDA	C=O	APX	CAT	SOD	GPX	GST	GSH
K	-0.84982	1																				
Mg	-0.82356	0.869732	1																			
P	-0.53477	0.407226	0.361676	1																		
N	-0.70804	0.477977	0.607211	0.614331	1																	
Ca	0.707626	-0.71072	-0.66916	-0.43059	-0.54255	1																
RGR fr	-0.63298	0.593545	0.484212	0.0081	0.057455	-0.38197	1															
RGR FW	-0.6884	0.638978	0.510908	0.245378	0.267604	-0.51254	0.906785	1														
Fv/Fm	-0.71772	0.677842	0.498378	0.511002	0.229402	-0.50357	0.257762	0.318186	1													
Φ_{PSII}	-0.67955	0.695045	0.779106	0.366016	0.517167	-0.59229	0.223196	0.250177	0.503131	1												
NPQ	0.475264	-0.20325	-0.17797	-0.4542	-0.3587	-0.04308	0.42243	0.265093	-0.50652	-0.35568	1											
Chl a	-0.44535	0.301759	0.312588	0.397345	0.539505	-0.5168	0.253127	0.459671	0.364037	0.3393	-0.28498	1										
Chl b	-0.14017	0.000699	-0.11687	0.299449	0.078935	-0.31294	0.00411	0.167876	0.50185	0.112338	-0.34256	0.74377	1									
Car	-0.42751	0.159739	0.10821	0.448955	0.377366	-0.16173	0.041661	0.20327	0.617275	0.113135	-0.63126	0.731531	0.781045	1								
MDA	0.770061	-0.5413	-0.72597	-0.36989	-0.81707	0.613579	-0.36697	-0.36151	-0.28198	-0.66374	0.048515	-0.45243	0.008198	-0.20281	1							
C=O	0.811921	-0.6818	-0.60302	-0.58501	-0.37417	0.63498	-0.4061	-0.46216	-0.85704	-0.41322	0.382946	-0.387	-0.39515	-0.56748	0.382692	1						
APX	0.528448	-0.60489	-0.77317	-0.12785	-0.36244	0.328938	-0.20459	-0.12235	-0.31411	-0.78267	0.198041	-0.17894	0.220713	0.063255	0.603822	0.180756	1					
CAT	0.865964	-0.78358	-0.68408	-0.6202	-0.4319	0.694075	-0.45871	-0.52642	-0.89046	-0.5313	0.348475	-0.3812	-0.35068	-0.51116	0.473887	0.977733	0.287589	1				
SOD	0.806683	-0.61971	-0.5007	-0.56581	-0.46203	0.629309	-0.41584	-0.50575	-0.81086	-0.49193	0.4124	-0.49433	-0.53443	-0.64577	0.461251	0.909027	0.077386	0.914025	1			
GPX	0.766529	-0.61959	-0.70362	-0.48265	-0.7673	0.670194	-0.58215	-0.60953	-0.41656	-0.7387	0.194645	-0.60491	-0.16009	-0.28521	0.90155	0.391483	0.715781	-0.49319	0.436036	1		
GST	0.838471	-0.73682	-0.78928	-0.47162	-0.61444	0.804768	-0.12253	-0.17628	-0.68811	-0.785	0.362557	-0.42884	-0.22366	-0.29685	0.711217	0.678236	0.666332	0.738789	0.614509	0.808516	1	
GSH	-0.7334	0.735668	0.810865	0.223382	0.326256	-0.55228	0.220417	0.125589	0.633422	0.634457	-0.21223	0.047303	-0.14468	0.050722	-0.55105	-0.60694	-0.76289	-0.66279	-0.401	-0.59165	-0.84953	1
ROS	0.537259	-0.06989	-0.10725	-0.84639	-0.62154	0.24567	-0.4822	-0.5695	-0.31805	-0.14234	0.354723	-0.28171	-0.2601	-0.46477	0.437922	0.445122	0.10475	-0.4469	0.508993	0.397894	0.314419	-0.05321

Numbers marked with red color are significant at $p < 0.05$.

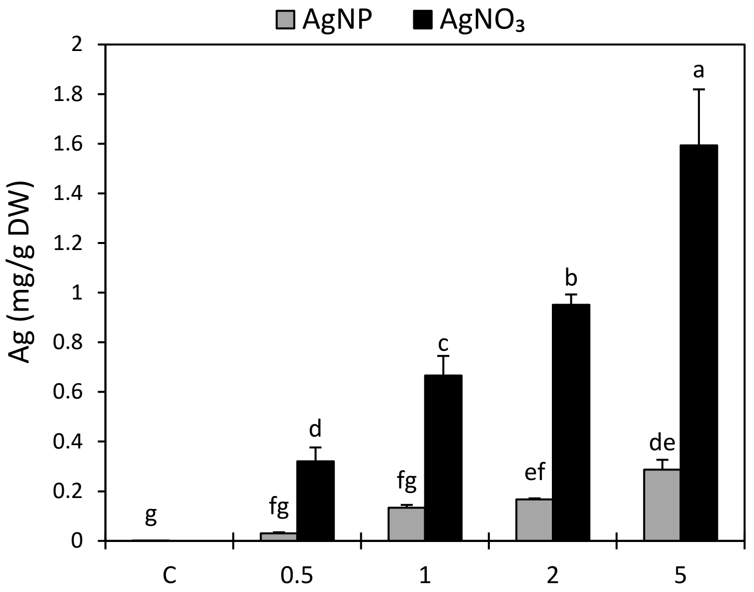

Figure S1. Ag content in duckweed after 7-day treatment with silver nanoparticles (AgNPs) and ionic silver ($AgNO_3$) in concentration range 0.5-5 mg/L or in control plants (C). Standard deviations are presented by error bars. Different letters indicate significantly different values at $P < 0.05$ according to ANOVA.
